# Blockade of multiple monoamines receptors reduce insulin secretion from pancreatic β-cells

**DOI:** 10.1038/s41598-019-52590-y

**Published:** 2019-11-11

**Authors:** Mao Nagata, Tomoharu Yokooji, Tomoe Nakai, Yumika Miura, Takashi Tomita, Takanori Taogoshi, Yumi Sugimoto, Hiroaki Matsuo

**Affiliations:** 1 Department of Pharmaceutical Services, Graduate School of Biomedical and Health Sciences, Hiroshima University, Hiroshima, Japan; 2 Department of Frontier Science for Pharmacotherapy, Graduate School of Biomedical and Health Sciences, Hiroshima University, Hiroshima, Japan; 3 Department of Pharmacology, Faculty of Pharmaceutical Sciences, Himeji Dokkyo University, Himeji, Japan

**Keywords:** Mechanisms of disease, Endocrine system and metabolic diseases, Pharmacology, Receptor pharmacology

## Abstract

Clinical use of olanzapine frequently causes severe hyperglycemia as an adverse effect. In this study, we elucidated mechanisms by which olanzapine reduced insulin secretion using the hamster pancreatic β-cell line HIT-T15. Reverse transcriptional-PCR analysis revealed expression of dopamine (D_2_, D_3_ and D_4_), serotonin (5-HT_2A_, 5-HT_2B_, 5-HT_2C_, and 5-HT_6_), and histamine (H_1_ and H_2_) receptors in HIT-T15 cells. Olanzapine decreased insulin secretion from HIT-T15 cells at clinically relevant concentrations (64–160 nM). A dopamine D_2_ agonist, D_3_ antagonist, and D_4_ antagonist suppressed insulin secretion, whereas a D_2_ antagonist and D_3_ agonist increased it. A serotonin 5-HT_2B_ agonist slightly increased insulin secretion, while a 5-HT_2C_ antagonist slightly decreased it. Other agonists and antagonists for serotonin receptors did not affect insulin secretion. A histamine H_1_ agonist increased insulin secretion, whereas an H_1_ antagonist and H_2_ agonist suppressed it. Our results suggest that dopamine (D_2_, D_3_ and D_4_), serotonin (5-HT_2B_ and 5-HT_2C_), and histamine (H_1_ and H_2_) receptors, which are expressed on pancreatic β-cells, directly modulate insulin secretion from pancreatic β-cells. Thus, olanzapine may induce hyperglycemia in clinical settings by suppressing insulin secretion from pancreatic β-cells through inhibition of dopamine D_3_, serotonin 5-HT_2B_ and 5-HT_2C_, and histamine H_1_ receptors.

## Introduction

Schizophrenia is a mental disorder that often appears in adolescence or early adulthood. According to an epidemiological study, the lifetime prevalence of schizophrenia is estimated at 0.7% worldwide^1^. Patients with schizophrenia typically present with various psychiatric symptoms including positive symptoms, negative symptoms, and cognitive symptoms, which result from dysregulated dopaminergic and non-dopaminergic modulation of the mesocorticolimbic system^2^. Positive symptoms such as delusions and hallucinations often develop during the acute phase and improve over time. In contrast, negative symptoms such as abulia, autism, dullness, and avolition, as well as cognitive deficits, generally become worse and chronic.

Pharmacotherapy with antipsychotic agents is a basic treatment for schizophrenia. Antipsychotic agents can be classified into two types: typical and atypical. Atypical antipsychotic agents such as risperidone, olanzapine, and aripiprazole are currently used as first-line agents to treat schizophrenia because they exhibit lower incidence of extrapyramidal symptoms and hyperprolactinemia compared with typical antipsychotic agents. Olanzapine, an atypical antipsychotic agent, exhibits effectiveness on positive symptoms, negative symptoms, and cognitive deficits by inhibiting multiple receptors such as dopamine, serotonin, histamine H_1_, α-adrenergic, and muscarinic acetylcholine receptors. According to a prescription survey conducted in 2013, olanzapine was the most frequently used among atypical antipsychotic drugs in Japan and Canada^3^. However, use of olanzapine has reportedly caused serious diabetic ketoacidosis and associated deaths as a result of hyperglycemia. In 2002, the Japan Ministry of Health, Labour, and Welfare issued Emergency Safety Information about the risk of serious hyperglycemia associated with olanzapine administration. However, mechanisms underlying olanzapine-induced hyperglycemia remain incompletely understood. Previous reports have suggested that repeated use of olanzapine caused increasing appetite, weight gain, and obesity, resulting in the development of type 2 diabetes^4,5^. However, olanzapine-induced hyperglycemia has been also observed in patients independent of weight gain^6^. Several reports have shown that olanzapine induced hyperglycemia by induction of apoptosis in insulin-secreting pancreatic β-cells^7^, insulin resistance^8^, increased glucose production in the liver^9^, and/or increased epinephrine secretion^10^. We hypothesized that olanzapine can increase hyperglycemia by suppressing insulin secretion from pancreatic β-cells via blockade of multiple monoamine receptors. In this study, we aimed to elucidate the involvement of dopamine, serotonin, and histamine receptors in insulin secretion from pancreatic β-cells.

## Results

### Expression of dopamine, serotonin, and histamine receptor mRNA in HIT-T15 cells

**Figure 1:**
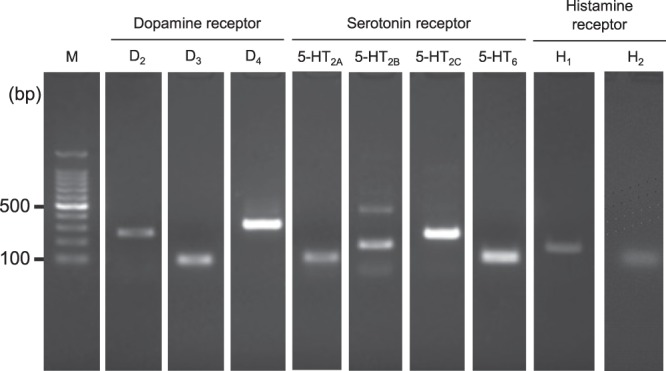
mRNA expression of dopamine (D_2_, D_3_, and D_4_), serotonin (5-HT_2A_, 5-HT_2B_, 5-HT_2C_, and 5-HT_6_), and histamine (H_1_ and H_2_) receptors in HIT-T15 cells. Total RNA extracted from HIT-T15 cells was reverse-transcribed, and first-strand cDNA was synthesized. Target genes were amplified with a set of specific primers (shown in Table 2). For expression analysis of dopamine D_3_ and D_4_, all serotonin, and histamine H_2_ receptors, two-step PCR was performed using nested primers (shown in Table 2). PCR products were separated by electrophoresis using a 2% agarose gel and stained with ethidium bromide. M, 100-bp ladder size marker.

Expression of dopamine (D_2_, D_3_, and D_4_), serotonin (5-HT_2A_, 5-HT_2B_, 5-HT_2C_, and 5-HT_6_), and histamine (H_1_ and H_2_) receptors were confirmed by reverse transcriptional (RT)-PCR analysis using specific primers and cDNA from hamster pancreatic cells (HIT-T15). A band corresponding to the expected size of each receptor was observed (Fig. 1).

### Olanzapine decreased insulin secretion from HIT-T15 cells

**Figure 2:**
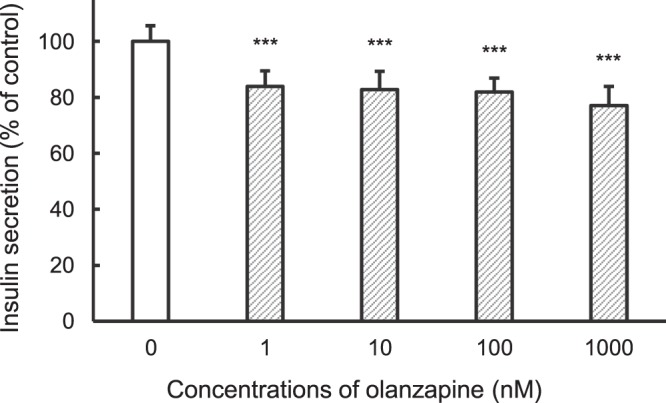
Effect of olanzapine on insulin secretion from HIT-T15 cells. HIT-T15 cells were incubated with medium containing 1% dimethylsulfoxide (control) or olanzapine for 1 h at 37 °C. Concentrations of insulin released into the medium were determined using a rat Insulin ELISA kit. Amounts of insulin secretion were normalized to the total protein content of each well. Each value represents mean ± SD of eight trials. ***P < 0.001 with respect to control.

Olanzapine decreased insulin secretion from HIT-T15 cells to ~80% of controls at concentrations of 1–1000 nM (Fig. 2). Notably, ~64–160 nM olanzapine has been observed in blood from patients orally administered olanzapine in clinical settings^11,12^.

### Effects of dopamine receptor agonists and antagonists on insulin secretion from HIT-T15 cells

**Figure 3:**
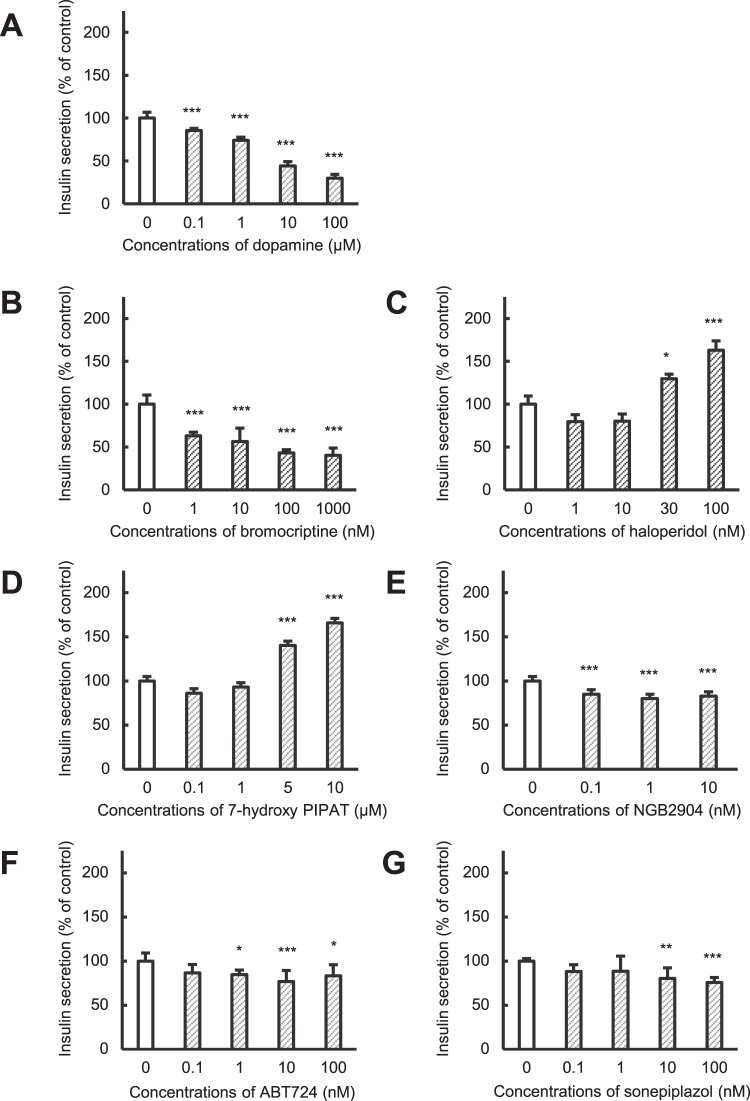
Effects of dopamine receptor agonists and antagonists on insulin secretion from HIT-T15 cells. HIT-T15 cells were incubated with medium containing 1% dimethylsulfoxide (control), dopamine (**A**), bromocriptine (**B**, D_2_ agonist), haloperidol (**C**, D_2_ antagonist), 7-hydroxy PIPAT (**D**, D_3_ agonist), NGB2904 (**E**, D_3_ antagonist), ABT724 (**F**, D_4_ agonist), or sonepiprazole (**G**, D_4_ antagonist) for 1 h at 37 °C. Concentrations of insulin released into the medium were determined using a rat Insulin ELISA kit. Amounts of insulin secretion were normalized to the total protein content of each well. Each value represents mean ± SD of four to eight trials. ***P < 0.001 with respect to control.

The involvement of dopamine receptors in insulin secretion was evaluated using HIT-T15 cells (Fig. 3). Dopamine decreased insulin secretion in a concentration-dependent manner (Fig. 3A), consistent with a previous report^13^. Bromocriptine, a dopamine D_2_ receptor agonist, decreased insulin secretion in a concentration-dependent manner and this decrease remained constant (~40% of control) at dosages over 100 nM (Fig. 3B). In contrast, haloperidol, a dopamine D_2_ receptor antagonist, increased insulin secretion (Fig. 3C). For the dopamine D_3_ receptor, the agonist 7-hydorxy PIPAT significantly enhanced insulin secretion (Fig. 3D), whereas the antagonist NGB2904 suppressed it (Fig. 3E). Furthermore, both the dopamine D_4_ receptor agonist ABT724 and antagonist sonepirazole significantly suppressed insulin secretion to ~80% of control (Fig. 3F,G). These findings suggest that stimulation of dopamine D_2_ and D_4_ receptors decreased insulin secretion from pancreatic β-cells, whereas stimulation of D_3_ receptors increased it.

### Effects of serotonin receptor agonists and antagonists on insulin secretion from HIT-T15 cells

**Figure 4:**
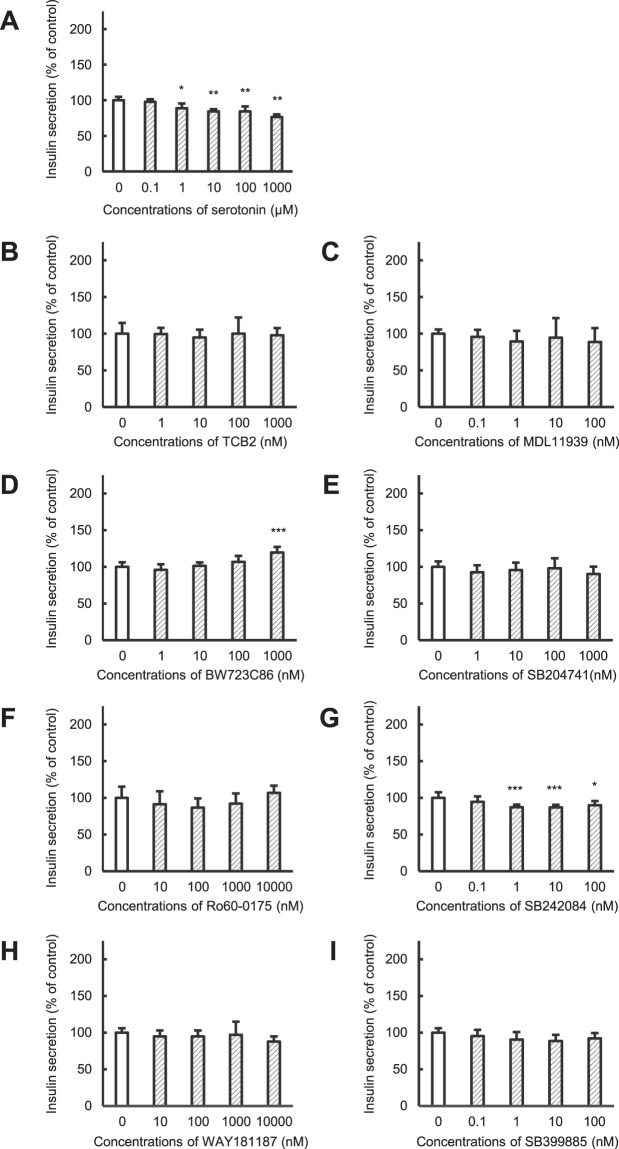
Effects of serotonin receptor agonists and antagonists on insulin secretion from HIT-T15 cells. HIT-T15 cells were incubated with medium containing 1% dimethylsulfoxide (control), serotonin (**A**), TCB2 (**B**, 5-HT_2A_ agonist), MDL11939 (**C**, 5-HT_2A_ antagonist), BW723C86 (**D**, 5-HT_2B_ agonist), SB204741 (E, 5-HT_2B_ antagonist), Ro60–0175 (**F**, 5-HT_2C_ agonist), SB242084 (**G**, 5-HT_2C_ antagonist), WAY181187 (**H**, 5-HT_6_ agonist), or SB399885 (**I**, 5-HT_6_ antagonist) for 1 h at 37 °C. Concentrations of insulin released into the medium were determined using a rat Insulin ELISA kit. Amounts of insulin secretion were normalized to the total protein content of each well. Each value represents mean ± SD of eight trials. *P < 0.05, **P < 0.01, ***P < 0.001 with respect to control.

The involvement of serotonin receptors in insulin secretion was evaluated using HIT-T15 cells (Fig. 4). Insulin secretion was decreased to ~80% of control by serotonin in a concentration-dependent manner (Fig. 4A). Neither the serotonin 5-HT_2A_ receptor agonist TCB2 nor antagonist MDL11939 had an effect on insulin secretion (Fig. 4B,C). The serotonin 5-HT_2B_ receptor agonist BW723C86 slightly increased insulin secretion (Fig. 4D), while the antagonist SB204741 exerted no significant effects on secretion (Fig. 4E). Stimulation of serotonin 5-HT_2C_ receptors by agonist Ro60–0175 did not affect insulin secretion, although blockade of serotonin 5-HT_2C_ receptors by antagonist SB242084 slightly decreased secretion (Fig. 4F). Similar to the serotonin 5-HT_2A_ receptor, neither the serotonin 5-HT_6_ receptor agonist WAY181187 or antagonist SB399885 had an effect on insulin secretion (Fig. 4G,H). These results suggest that stimulation of serotonin 5-HT_2B_ and 5-HT_2C_ receptors can increase insulin secretion from pancreatic β-cells, although their contributions are much lower than those of dopamine receptors.

### Effects of histamine receptor agonists or antagonists on insulin secretion from HIT-T15 cells

**Figure 5:**
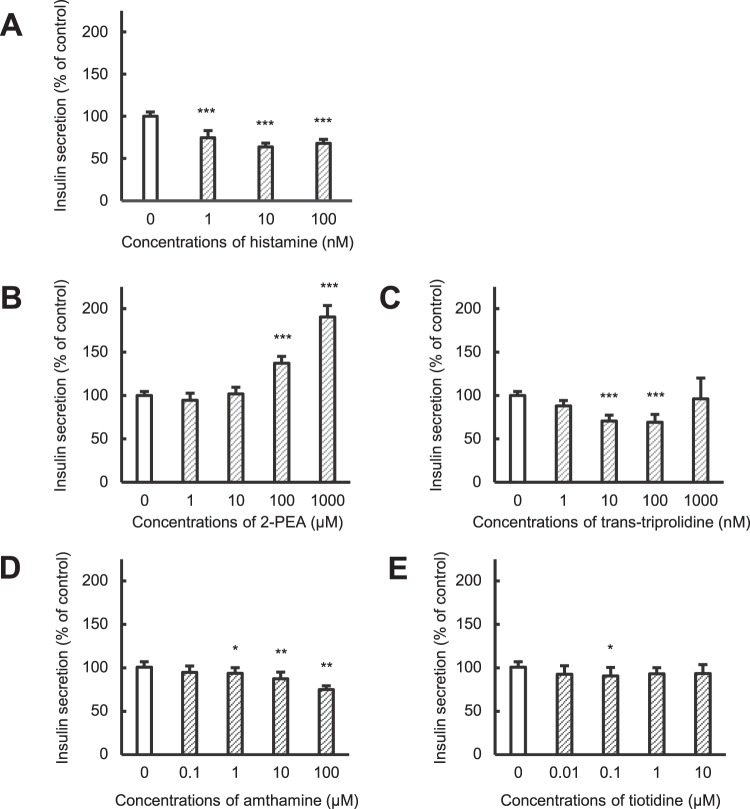
Effects of histamine receptor agonists and antagonists on insulin secretion from HIT-T15 cells. HIT-T15 cells were incubated with medium containing 1% dimethylsulfoxide (control), histamine (**A**), 2-pyridylethylamine (2-PEA) (**B**, H_1_ agonist), trans-triprolidine (**C**, H_1_ antagonist), amthamine (**D**, H_2_ agonist), or tiotidine (**E**, H_2_ antagonist) for 1 h at 37 °C. Concentrations of insulin released into the medium were determined using a rat Insulin ELISA kit. Amounts of insulin secretion were normalized to the total protein content of each well. Each value represents mean ± SD of eight trials. ***P < 0.001 with respect to control.

We evaluated the involvement of histamine receptors in insulin secretion from HIT-T15 cells (Fig. 5). Histamine decreased insulin secretion at 1–100 nM (Fig. 5A). The histamine H_1_ receptor agonist 2-pyridylethylamine (2-PEA) increased insulin secretion in a concentration-dependent manner, whereas trans-triprolidine, a histamine H_1_ receptor antagonist, decreased secretion to ~40% of control (Fig. 5B,C). In contrast, the histamine H_2_ receptor agonist amthamine decreased insulin secretion to ~75% of control in a concentration-dependent manner. In addition, the histamine H_2_ receptor antagonist tiotidine slightly decreased insulin secretion (Fig. 5D,E). Thus, stimulation of histamine H_1_ receptors increased insulin secretion from pancreatic β-cells, whereas stimulation of H_2_ receptors decreased secretion.

### Olanzapine nor receptor agonists or antagonists affected the viability of HIT-T15 cells

To evaluate whether cytotoxic effects of olanzapine and agonists or antagonists for each receptor occurred, an XTT assay was performed. As shown in Table 1, 1-h exposure of each agent tested did not affect the viability of HIT-T15 cells, indicating that alterations in insulin secretion from HIT-T15 cells induced by these compounds were not the result of cytotoxicity.

## Discussion

Several reports have shown that administration of olanzapine could induce hyperglycemia^14,15^. As olanzapine exerts an antipsychotic effect by inhibiting multiple receptors for dopamine, serotonin, histamine, adrenaline, and acetylcholine, we hypothesized that olanzapine could increase hyperglycemia by suppressing insulin secretion from pancreatic β-cells through blockade of multiple receptors. In this study, we investigated the involvement of dopamine, serotonin, and histamine receptors in insulin secretion using HIT-T15 cells.

The effects of olanzapine on plasma insulin levels are incompletely understood because doses of olanzapine used in previous animal experiments were higher than used in clinical settings. Nagata *et al*.^10^ reported that serum concentrations of insulin increased following a single intravenous infusion of olanzapine at a dose of 2.5–10 mg/kg. In their study, serum concentrations of olanzapine were 394–2763 ng/mL (1.26–8.84 µM) at 15 min after olanzapine administration. Simpson *et al*.^13^ reported that olanzapine increased insulin secretion from human islets at concentrations of 1–5 µM. In clinical settings, olanzapine is administered orally at a dose of 5–20 mg daily, yielding reportedly therapeutic serum concentrations of 20–50 ng/mL (64–160 nM)^12^. In this study, olanzapine decreased insulin secretion from HIT-T15 cells by ~20% compared with controls at concentrations of 1–1000 nM (Fig. 2). Our preliminary study showed that olanzapine increased insulin secretion from HIT-T15 cells at concentrations of 10–30 µM (data not shown). These findings suggest that olanzapine directly suppresses insulin secretion from pancreatic β-cells at clinical concentrations. Blood glucose levels were reportedly altered by 20% when insulin secretion was altered by 25% after feeding^16^, suggesting that olanzapine can induce hyperglycemia by suppressing insulin secretion from pancreatic β-cells at clinical concentrations.

We demonstrated that dopamine D_2_, D_3_, and D_4_; serotonin 5-HT_2A_, 5-HT_2B_, 5-HT_2C_, and 5-HT_6_; and histamine H_1_ and H_2_ receptors are expressed by HIT-T15 cells (Fig. 1). A few previous studies reported expression of these receptors in human pancreas^17–19^. Rubí *et al*.^17^ reported the detection of dopamine D_2_ and D_4_ receptor mRNAs in human islets. Bonhaus *et al*.^18^ observed mRNAs for serotonin 5-HT_2A_, 5-HT_2B_, and 5-HT_2C_ receptors in human pancreas. Although no previous studies reported expression of histamine H_1_ or H_2_ receptors in human pancreas, Szukiewicz *et al*.^19^ reported protein expression of these receptors in pancreatic β-like cells differentiated from human amniotic epithelial cells by nicotinamide treatment. Thus, the involvement of these receptors in insulin secretion may be observed even in human pancreas.

To evaluate the involvement of dopamine, serotonin, and histamine receptor subtypes in insulin secretion, we examined the effects of agonists and antagonists specific for each receptor subtype on insulin secretion from HIT-T15 cells. With regard to dopamine receptors, stimulation of the dopamine D_2_ receptor suppressed insulin secretion, whereas its blockade enhanced it (Fig. 3B,C). This result is consistent with a previous report that blockade of dopamine D_2_ receptor enhanced insulin secretion from human islets^13^. In contrast, stimulation of the dopamine D_3_ receptor enhanced insulin secretion, whereas its blockade enhanced secretion (Fig. 3D,E). Thus, olanzapine can suppress insulin secretion via blockade of the dopamine D_3_ receptor. Insulin secretion was increased by either stimulation or blockade of the dopamine D_4_ receptor (Fig. 3F,G) using specific dopamine D_4_ agonist ABT724 (EC_50_ value for rat dopamine D_4_ receptor is 12.4 nM and that for dopamine D_2_ is >10 µM) or antagonist sonepiprazole (K_i_ value for human dopamine D_4_ receptor is 10 nM and those for other monoamine receptors are >2 µM), respectively, which seems contradictory^20,21^. However, these findings can be explained by differences in expression levels between dopamine D_2_ and D_3_ receptors. Dopamine can stimulate both dopamine D_2_ and D_3_ receptors when the dopamine D_4_ receptor is blocked. Expression levels of dopamine D_2_ receptor were higher than those of dopamine D_3_ receptor, as expression of mRNA for dopamine D_3_ could be detected by two-step PCR with nested primers (Fig. 1). We considered decreased insulin secretion via blockade of the dopamine D_4_ receptor to arise from stimulation of the dopamine D_2_ receptor.

For serotonin receptors, stimulation of the 5-HT_2B_ receptor slightly increased insulin secretion from HIT-T15 cells, whereas blockade of the 5-HT_2C_ receptor decreased secretion (Fig. 4C,F). In contrast, a 5-HT_2B_ antagonist, 5-HT_2C_ agonist, and both an agonist and antagonist of 5-HT_2A_ and 5-HT_6_ did not affect insulin secretion. Bennet *et al*.^22^ demonstrated that stimulation of 5-HT_2B_ receptor increased the glucose-stimulated insulin secretion from mouse and human pancreatic β-cells by triggering downstream changes in cellular Ca^2+^ flux that enhance mitochondrial metabolism. These findings suggest that serotonin 5-HT_2B_ and 5-HT_2C_ receptors can modulate insulin secretion from β-cells. Thus, inhibition of serotonin 5-HT_2B_ and 5-HT_2C_ receptors may be involved in olanzapine-reduced insulin secretion, although their contributions may be less than those of dopamine receptors. Interestingly, there is one previous reports showing that 5-HT_3_ receptor-mediated insulin secretion was further enhanced in pregnant mice compared to that in normal mice^23^. Thus, it is possible that the contributions of serotonin receptors subtypes to insulin secretion are altered under diseased states.

We also evaluated the involvement of histamine receptors in insulin secretion (Fig. 5). Histamine decreased insulin secretion at 1–100 nM (Fig. 5A). Stimulation of the histamine H_1_ receptor increased insulin secretion, whereas stimulation of the histamine H_2_ receptor decreased it (Fig. 5B,D). Thus, insulin secretion from pancreatic β-cells can be modulated by both histamine receptor subtypes, and olanzapine can suppress insulin secretion via blockade of the histamine H_1_ receptor.

The roles of endogenous monoamines in the insulin secretion have not been understood completely. Ustione and Piston^24^ reported that dopamine was secreted from pancreatic β-cells simultaneously with insulin and caused negative feedback inhibition on insulin secretion. In agreement with their report, we also showed that physiological levels of dopamine suppressed the insulin secretion from pancreatic β-cells (Fig. 3). In contrast, there have been no reports regarding the roles of endogenous serotonin and histamine on insulin secretion. In this study, we used dopamine, serotonin and histamine at concentrations of 0.1–100 µM, 0.1–100 µM and 1–100 nM, respectively. These concentrations of dopamine and serotonin are higher than those in physiological concentrations which in human plasma are reportedly ~6.5 nM^25^ and ~0.6 pM^26^, respectively. Thus, further studies are necessary to understand the role of endogenous monoamines in the insulin secretion at physiological conditions.

In this study, we did not confirm the expression and involvement of muscarinic acetylcholine receptors on insulin secretion from HIT-T15 cells. Iismaa *et al*.^27^ reported that muscarinic acetylcholine receptors were expressed on rat pancreatic β-cells. Furthermore, Henquin *et al*.^28^ reported that insulin secretion was enhanced via stimulation of muscarinic receptors. Because olanzapine inhibits the muscarinic acetylcholine receptors, it is speculated that olanzapine can suppress the insulin secretion from pancreatic β-cells via blockade of muscarinic acetylcholine receptors as well as monoamine receptors. Further studies are necessary to clarify the involvement of muscarinic acetylcholine receptors in insulin secretion from pancreatic β-cells.

In conclusion, we demonstrated that olanzapine suppressed insulin secretion from pancreatic β-cells via blockade of dopamine D_3_, serotonin 5-HT_2B_ and 5-HT_2C_, and histamine H_1_ receptors at clinical concentrations *in vitro*. Although further studies are necessary using human pancreatic β-cells for *in vitro* and *in vivo* animal studies, these findings shed new light on the mechanisms underlying olanzapine-induced hyperglycemia.

## Materials and Methods

### Chemicals

Olanzapine and haloperidol were obtained from FUJIFILM Wako Pure Chemical Corporation (Osaka, Japan). Dopamine hydrochloride and bromocriptine were purchased from Sigma-Aldrich (St Louis, MO, USA). 7-Hydroxy PIPAT, ABT724, TCB2, BW723C86, Ro60–0175, WAY181187, 2-PEA, NGB2904, sonepiprazole, MDL11939, SB204741, SB399885, trans-triprolidine, amthamine, and tiotidine were from Tocris Bioscience (Bristol, England, UK). SB242084 was obtained from Toronto Research Chemicals (Ontario, Canada). All other chemicals used were of the highest purity available.

### Cell culture

HIT-T15 cells were obtained from Sumitomo Dainippon Pharma (Osaka, Japan). Cells were cultured in Ham’s F12K medium (Sigma-Aldrich) containing 10% fetal bovine serum, 100 units/mL penicillin G, 100 µg/mL streptomycin, and 10 mM glucose which corresponds to the physiological blood concentrations in human in an atmosphere of 5% CO_2_/95% air at 37 °C. Cells were subcultured once a week using 0.25% EDTA and 0.038% trypsin. Fresh medium was replaced every 2 days. Cells were used between passages 80 and 100.

### RT-PCR analysis

Total RNA was extracted from HIT-T15 cells using an RNeasy Plus Mini Kit (Qiagen, Hilden, Germany) according to the manufacturer’s instructions. Next, total RNA was used for reverse transcription to synthesize cDNA using a ReverTra Ace qPCR RT kit (Qiagen). PCR was performed with an iCycler (Bio-Rad Laboratories, Hercules, CA, USA) using KOD-Plus-DNA polymerase (Toyobo, Osaka, Japan). Conditions for PCR were as follows: initial denaturation at 94 °C for 2 min; denaturation at 94 °C for 30 sec; annealing at optimal temperatures for dopamine, serotonin, and histamine receptors for 30 sec; and extension at 68 °C for 1 min (35 cycles). Primers, annealing temperatures, and product sizes for each receptor are summarized in Table 2. To examine expression of mRNA for dopamine D_3_ and D_4_ receptors, and all serotonin receptors, we performed two-step PCR with nested primers due to their lower expression in HIT-T15 cells. Nested primers for each receptor are summarized in Table 2. Conditions for the second round of PCR were the same as those for the first round. PCR products were electrophoresed with a 2% agarose gel and visualized under ultraviolet light with ethidium bromide.

**Table 1:** Effects of chemicals on HIT-T15 cell viability.

Chemical	Cell Viability (% of control)
olanzapine	101.2 ± 9.1
bromocriptine	108.2 ± 13.1
haloperidol	105.1 ± 4.7
7-hydroxy PIPAT	94.9 ± 4.1
NGB2904	106.4 ± 3.3
ABT724	104.7 ± 3.3
sonepiprazole	99.7 ± 8.1
TCB2	101.0 ± 9.1
MDL11939	121.4 ± 23.9
BW723C86	94.7 ± 7.9
SB204741	103.2 ± 10.9
Ro60–0175	103.2 ± 16.0
SB242084	99.8 ± 5.0
WAY181187	95.6 ± 12.5
SB39985	113.4 ± 8.6
2-pyridylethylamine	105.2 ± 7.2
trans-triprolidine	102.7 ± 3.4
amthamine	89.1 ± 21.2
tiotidine	93.5 ± 17.2

### Insulin secretion assay

Insulin secretion assays were performed according to previous reports^29,30^. Briefly, HIT-T15 cells were seeded at a density of 1.0 × 10^5^ cells/well in 24-well plates and cultured for 72 h after seeding. Next, cells were pre-incubated with fresh medium containing 1% dimethylsulfoxide (DMSO) for 30 min at 37 °C. After pre-incubation, cells were incubated with fresh medium for 1 h at 37 °C. To examine the effects of olanzapine or agonists/antagonists for each receptor on insulin secretion, each compound was added to the medium at various concentrations during incubation. Compounds tested are shown in Table 3. After incubation, the concentration of insulin released into the medium was determined using a rat Insulin ELISA kit (Morinaga Institute of Biological Science, Yokohama, Japan) according to our previously reported method^31,32^. Next, residual cells were washed with phosphate-buffered saline (pH 7.4), and lysed with 0.3 M NaOH. Concentrations of total protein were determined by Lowry method with bovine serum albumin as the standard. Amounts of insulin secretion were normalized to the total protein content of each well.

**Table 2:** Primer sequences, annealing temperatures, and product sizes.

Gene	Primer sequence	Annealing temperature (°C)	Product size (bp)
dopamine D_2_	forward: 5′-TCGCCATTTGTCTGGGTCCTG-3′	65	261
	reverse: 5′-TGCCCTTTGAGGGGGGTCTTC-3′		
dopamine D_3_ (1^st^ PCR)	forward: 5′-GTCTGGAATTTCAGCCGCATTTGCTGTGA -3′	62	119
	reverse: 5′-ATGACCACTGCTGTGTACCTGTCTATGCTG-3′		
(2^nd^ PCR)	forward: 5′-CAGCCGCATTTGCTGTGATG-3′	62	94
	reverse: 5′-GTACCTGTCTATGCTGATGGCA-3′		
dopamine D_4_ (1^st^ PCR)	forward: 5′-GTCCGCTCATGCTACTGCT-3′	60	344
	reverse: 5′-GACTCTCATTGCCTTGCGCTC-3′		
(2^nd^ PCR)	forward: 5′-GCTACTGCTTTACTGGGCCAC-3′	60	329
	reverse: 5′-TCATTGCCTTGCGCTCCCTT-3′		
serotonin 5-HT_2A_ (1^st^ PCR)	forward: 5′-CTGGTCATCATGGCAGTGTCCCTAGAGAA-3′	67	291
	reverse: 5′-GGTTCTGGAGTTGAAGCGGCTATGGTGGA-3′		
(2^nd^ PCR)	forward: 5′-TGATGTCACTTGCCATAGCTG-3′	55	105
	reverse: 5′-AGAGCTTGCTGGGCAAAG-3′		
serotonin 5-HT_2B_ (1^st^ PCR)	forward: 5′-ATGCCGATTGCCCTCTTGAC-3′	67	185
	reverse: 5′-CGGGAGTTGCACTGATTGG-3′		
(2^nd^ PCR)	forward: 5′-GCCGATTGCCCTCTTGACA-3′	62	182
	reverse: 5′-GGGAGTTGCACTGATTGGC-3′		
serotonin 5-HT_2C_ (1^st^ PCR)	forward: 5′-GGGTCCTTCGTGGCATTCTTCATCCCG-3′	65	273
	reverse: 5′-CTTTTCGTTGTTGATAGCTTGCATGGTGCC-3′		
(2^nd^ PCR)	forward: 5′-GTGGCATTCTTCATCCCGTTG-3′	62	254
	reverse: 5′-TTGATAGCTTGCATGGTGCT-3′		
serotonin 5-HT_6_ (1^st^ PCR)	forward: 5′-ATGCTGAACGCGCTGTATGG-3′	60	140
	reverse: 5′-GAGAGGATGAGCAGGTAGCG-3′		
(2^nd^ PCR)	forward: 5′-GTATGGGCGCTGGGTGCTA-3′	60	112
	reverse: 5′-GTAGCGGTCCAGGCTGATG-3′		
histamine H_1_	forward: 5′-ACTTGAACCGAGAGCGGAAG-3′	60	178
	reverse: 5′-GGGTTCAGCGTGGAGTTGAT-3′		
histamine H_2_ (1^st^ PCR)	forward: 5′-CCAGCTCCTGTGACTCCAGA-3′	60	353
	reverse: 5′-GGGTTTGGGAAGGTCTGATG-3′		
(2^nd^ PCR)	forward: 5′-GATCCCTTGCACAAACCCAAC-3′	60	97
	reverse: 5′-TCCTGGTCTGTAGTGTGCGT-3′

**Table 3:** Agonists and antagonists specific for dopamine, serotonin, or histamine receptors used in this study.

Receptor	Agonist	Antagonist
dopamine D_2_	bromocriptine	haloperidol
dopamine D_3_	7-hydroxy PIPAT	NGB2904
dopamine D_4_	ABT724	sonepiprazole
serotonin 5-HT_2A_	TCB2	MDL11939
serotonin 5-HT_2B_	BW723C86	SB204741
serotonin 5-HT_2C_	Ro60–0175	SB242084
serotonin 5-HT_6_	WAY181187	SB399885
histamine H_1_	2-pyridylethylamine	trans-triprolidine
histamine H_2_	amthamine	tiotidine

### XTT assay

HIT-T15 cells were seeded at a density of 1.5 × 10^4^ cells/well in 96-well plates and cultured for 24 h. Next, cells were replaced with serum-free Ham’s F12K medium containing optimal concentrations of olanzapine, an agonist or antagonist of each receptor, or 1% DMSO (control). Cells were incubated for 1 h at 37 °C. After washing, 200 µL of Hank’s Balanced Salt Solution containing 225 µM XTT and 48 µM 1-methoxy-PMS was added to each well. After incubation for 4 h at 37 °C, absorbance was measured at 450 nm against 630 nm as a reference using a Multiskan GO (Thermo Fisher Scientific, Waltham, MA, USA).

### Statistical analyses

Data are displayed as mean ± standard deviation of the mean (SD). Differences in mean values between groups were assessed using Kruskal-Wallis or ANOVA tests, followed by post hoc Tukey test, Dunnett’s test, Bonferroni test, or Student’s t-test. P < 0.05 was considered statistically significant.
